# Peri-coronary fat attenuation index combined with high-risk plaque characteristics quantified from coronary computed tomography angiography for risk stratification in new-onset chest pain individuals without acute myocardial infarction

**DOI:** 10.1371/journal.pone.0304137

**Published:** 2024-05-28

**Authors:** Xuelong Zhang, Zelong Cao, Jianan Xu, Xing Guan, Honghou He, Linan Duan, Lishuang Ji, Gang Liu, Qifeng Guo, Yang You, Mingqi Zheng, Mei Wei

**Affiliations:** 1 The First Hospital of Hebei Medical University, Shijiazhuang, Hebei, China; 2 Graduate School of Hebei Medical University, Shijiazhuang, Hebei, China; 3 Hebei Key Laboratory of Heart and Metabolism, Shijiazhuang, Hebei, China; Universitatsklinikum Wurzburg, GERMANY

## Abstract

This study aims to evaluate the role of the peri-coronary Fat Attenuation Index (FAI) and High-Risk Plaque Characteristics (HRPC) in the assessment of coronary heart disease risk. By conducting coronary CT angiography and coronary angiography on 217 patients with newly developed chest pain (excluding acute myocardial infarction), their degree of vascular stenosis, FAI, and the presence and quantity of HRPC were assessed. The study results demonstrate a correlation between FAI and HRPC, and the combined use of FAI and HRPC can more accurately predict the risk of major adverse cardiovascular events (MACE). Additionally, the study found that patients with high FAI were more prone to exhibit high-risk plaque characteristics, severe stenosis, and multiple vessel disease. After adjustment, the combination of FAI and HRPC improved the ability to identify and reclassify MACE. Furthermore, the study identified high FAI as an independent predictor of MACE in patients undergoing revascularization, while HRPC served as an independent predictor of MACE in patients not undergoing revascularization. These findings suggest the potential clinical value of FAI and HRPC in the assessment of coronary heart disease risk, particularly in patients with newly developed chest pain excluding acute myocardial infarction.

## 1. Introduction

Cardiovascular disease is currently one of the leading causes of morbidity and mortality worldwide, with approximately 7.4 million deaths attributed to coronary heart disease (CHD) each year [1]. Factors such as population aging, lifestyle changes, and increased awareness of personal health status have led to a year-on-year increase in the incidence of coronary heart disease worldwide, posing a significant public health issue threatening the lives and well-being of the middle-aged and elderly population. CHD, which is a common type of organ damage resulting from atherosclerosis, poses a significant threat to human health. Inflammation plays a crucial role not only in the development and progression of atherosclerosis but also in the rupture of unstable plaques [2], which can lead to acute cardiovascular events. Therefore, early and accurate assessment of coronary vascular inflammation can effectively prevent adverse cardiovascular events. However, the currently available methods for detecting vascular inflammation, such as inflammatory biomarkers like high-sensitivity C-reactive protein and pro-inflammatory cytokines, mainly reflect systemic inflammation and fail to accurately identify coronary inflammation and vulnerable atherosclerotic plaques [3–6]. Invasive techniques like intravascular ultrasound and optical coherence tomography have limited specificity in reflecting coronary vascular inflammation. Through the in-depth analysis of the existing non-invasive imaging techniques such as positron emission tomography-computed tomography (PET-CT) and magnetic resonance imaging (MRI), it can be seen that they have some limitations in the diagnostic process, such as high cost, large radiation dose, and complex examination process. In contrast, FAI and HRPC, as new evaluation methods, have lower cost, less radiation exposure and simpler operation process, which may bring better medical experience to patients [7].Therefore, there is a need for convenient and cost-effective non-invasive imaging methods to detect coronary vascular inflammation, which is worth further exploration in the field of cardiovascular research.

Studies have shown that peri-coronary fat is closely associated with coronary artery vascular inflammation and is involved in the development of atherosclerosis [8]. The composition of peri-coronary fat tissue can be altered based on the degree of vascular inflammation. The change in CT attenuation values can serve as a tool to assess the level of coronary artery inflammation. In recent years, a non-invasive imaging biomarker for detecting vascular inflammation, called the Fat Attenuation Index (FAI) of peri-coronary fat, has emerged. FAI is derived from the analysis of three-dimensional changes in CT attenuation of peri-vascular adipose tissue (PVAT) surrounding the diseased coronary artery segment using coronary artery CT angiography (CCTA) [9]. Researchers determine the average attenuation coefficient within a predefined radiodensity window (−190 to −30 HU) using CCTA. When inflammation occurs in the coronary artery, there is a shift in attenuation from the adipose phase (more negative Hounsfield units [HU] values, such as close to -190 HU) to the aqueous phase (less negative HU values, such as close to -30 HU). Previous studies have shown that FAI can more effectively detect inflammation of the coronary arteries at an early stage, which is of significant importance for the risk stratification of cardiovascular disease patients and the identification of individuals at high risk of future cardiovascular events. It also serves as an independent predictive indicator for the occurrence of adverse cardiovascular events [8].

At the current stage, coronary CT angiography (CCTA), as a highly sensitive non-invasive imaging method for the diagnosis of coronary heart disease, has gained wider application in clinical medical practice. Compared to invasive coronary angiography, coronary CTA is better suited for evaluating and guiding further treatment in patients with new-onset chest pain without acute myocardial infarction. In the United Kingdom alone, the number of annual coronary CTA scans may exceed 350,000 [10], while in China, the Chinese Science Bulletin reports that more than 5 million people undergo vascular CT angiography scans each year. Previous studies have demonstrated that CTA not only allows analysis of the coronary artery surrounding adipose tissue attenuation index (FAI), but also enables accurate assessment of plaque characteristics, such as positive remodeling, spotty calcifications, low-attenuation plaques, and napkin-ring sign [11]. Researchers have shown through histological and intravascular imaging methods that these plaque features represent high-risk plaques [12]. Moreover, the presence of these high-risk plaque characteristics is closely associated with the occurrence of major adverse cardiovascular events [13]. Invasive and pathological studies have indicated that patients with high-risk plaques are more likely to experience acute coronary syndrome and sudden cardiac death [14].

Although both the peri-coronary Fat Attenuation Index (FAI) and high-risk plaques are independent predictors of major adverse cardiovascular events in patients with cardiovascular disease, further research is needed to determine whether the presence of FAI provides additional predictive value for risk assessment in patients with high-risk plaques, especially for the convenient evaluation of new-onset chest pain in patients without acute myocardial infarction. Furthermore, more evidence is required to verify the relationship and effects of FAI on high-risk plaques in order to promote its widespread application in clinical practice.

The aim of this study is to investigate the correlation between FAI and HRPC, as well as the combined predictive value and mutual influence of these two factors on prognosis in patients with new-onset chest pain without acute myocardial infarction. The study particularly focuses on examining the differential effects of FAI and HRPC on the decision of whether to perform revascularization procedures.

## 2. Materials and methods

### 2.1 Study population

We conducted a study on patients with coronary artery disease (CAD) who visited our cardiology department from June 2020 to June 2022. A total of 217 patients were screened. All patients underwent coronary computed tomography angiography (CCTA) and coronary angiography (CAG). Inclusion criteria were: (1) aged ≥ 18 years; (2) newly diagnosed angina or chest pain. Exclusion criteria were: (1) history of previous angina or chest pain; (2) acute myocardial infarction; (3) patients with indeterminate image quality on CTA; (4) patients with CTA contrast agent allergy; (5) patients with valvular heart disease, dilated cardiomyopathy, or severe heart failure; (6) patients with stage 5 chronic kidney disease.

Coronary CTA is part of routine clinical practice for suspected coronary artery disease patients, and the decision to proceed with coronary CTA before invasive angiography is made by the attending physician. The enrolled patients did not participate in conflicting studies. The study was performed according to the guidelines of the Helsinki Declaration and has been approved by the ethics committees at the First Hospital of Hebei Medical University, China (20220362). Since data were evaluated pseudonymously and were solely obtained for treatment purposes, a requirement of informed consent was waived by the Ethics Committee of the First Hospital of Hebei Medical University.

### 2.2 Analysis of coronary artery CTA and high-risk plaque characteristics and FAI measurement

Coronary CTA images were obtained following the guidelines of the Society of Cardiovascular Computed Tomography for coronary CTA performance, using a 320-detector-row scanning platform (Siemens Healthcare GmbH, Henkestr. 127, 91052 Erlangen, Germany) and post-processing software: Shukun Coronary CTA Intelligent Diagnostic Software. The coronary CTA images were analyzed in a blinded manner at the CT room (Hebei Medical University First Hospital, Hebei, China) [15]. Patients with a heart rate greater than 65 beats per minute (bpm) received oral administration of 25–75 mg β-blockers one hour before the scan. The contrast agent dosage was determined based on the patient’s weight and scan time.

Qualitative analysis of high-risk plaque characteristics was performed based on previous studies [16]. Plaque density was evaluated semi-automatically using a dedicated cardiac workstation. The presence of the following high-risk plaque characteristics was analyzed in the CT room: (1) low-attenuation plaque: average density < 30 Hounsfield units (HU), measured from three random regions of interest within the non-calcified low-attenuation plaque area of approximately 0.5–1.0 mm^2^, which is closely associated with the necrotic core in atherosclerotic plaques confirmed by intravascular ultrasound (IVUS); (2) positive remodeling: involves compensatory enlargement of the vessel wall in the atherosclerotic plaque area to maintain the area of the lumen unchanged. Once the remodeling index reaches or exceeds the standard of 1.1, we can determine that positive remodeling has occurred; (3) napkin-ring sign: characterized by the CT attenuation difference between a large necrotic core (central low-attenuation) and fibrous plaque tissue (circular high attendant)); (4)spotty calcification: small and dense plaque components surrounded by non-calcified plaque tissue (average density > 130 HU, diameter < 3 mm, calcium length < 1.5 times the vessel diameter, and calcium point width less than two-thirds of the vessel diameter).

To precisely measure the perivascular FAI, we tracked three major epicardial coronary vessels (right coronary artery, left anterior descending artery, and left circumflex artery) and defined the perivascular fat around each vessel as the radial distance from the outer vessel wall equal to the vessel diameter. To avoid the influence of the aortic wall, we excluded the proximal 10mm of the right coronary artery and analyzed the proximal 10-50mm segment [8], and for the left anterior descending artery and left circumflex artery, we analyzed the proximal 40mm of each vessel. We did not analyze the left main coronary artery due to its variable length. Previous studies [17, 18] have indicated that the average FAI of the three major epicardial vessels one month after percutaneous coronary intervention for acute coronary syndrome (ACS) is the only meaningful predictor of coronary flow reserve. Considering the multicollinearity of the FAI values for the three coronary arteries and the grouping of this study by individuals rather than by vessels, the average FAI value of the three vessels was used to represent the overall inclusion in the analysis.

### 2.3 Coronary angiography

The angiographic data were obtained from the records of the catheterization laboratory. Coronary angiography was performed by three dedicated interventional physicians, who chose between radial or femoral artery access based on their discretion. Coronary angiography was used to assess the severity of coronary artery disease. Coronary artery stenosis ≥50% was defined as coronary heart disease, and the complexity of coronary atherosclerosis in patients was quantified using the Gensini score. To aid in the generation of the Gensini score, each patient obtained at least five different angiographic views. In this study, the traditional Gensini coronary artery stenosis scoring system was employed [19], with the most severe stenosis within each coronary artery vessel used to determine the degree of stenosis for that vessel. The specific scoring criteria were as follows: a stenosis of 0–25% was assigned a score of 1; a stenosis of 25–50% was assigned a score of 2; a stenosis of 50–75% was assigned a score of 4; a stenosis of 75–90% was assigned a score of 8; a stenosis of 90–99% was assigned a score of 16; and a stenosis exceeding 99% was assigned a score of 32. Since different coronary arteries play varying roles in myocardial blood supply, we applied coefficients to account for these differences and calculate the score for each vessel in each patient. The coefficients used were: 5 for left main disease, 2.5 for proximal left anterior descending disease, 1.5 for mid left anterior descending disease, 1 for distal left anterior descending disease, 1 for first diagonal disease, 0.5 for second diagonal disease, 2.5 for proximal left circumflex disease, 1 for mid and distal left circumflex and obtuse marginal disease, 0.5 for posterolateral disease, and 1 for proximal, mid, distal, and posterolateral branches of the right coronary artery disease. By employing the Gensini method described above and summing the scores of each coronary artery, including its branches, we arrived at the total score, which represents the overall stenosis severity.

### 2.4 Blood sample collection and analysis

A total of 4ml of venous blood was collected on the morning after admission. The blood samples were centrifuged at 3000r/min, and the serum was separated and stored at -20°C for further analysis. Various parameters of interest were measured for all patients who met the study criteria.

### 2.5 Clinical data and grouping

Gender, age, body mass index (BMI), risk factors, subsequent revascularization treatments, and baseline biochemical indicators were recorded. Risk factors included hypertension, diabetes, family history of coronary heart disease, smoking history, alcohol consumption history, and history of cerebrovascular disease. Biochemical indicators included total cholesterol (TC), triglycerides (TG), high-density lipoprotein cholesterol (HDL), low-density lipoprotein cholesterol (LDL), creatinine, uric acid, apolipoprotein A1, apolipoprotein B, lipoprotein(a), absolute neutrophil count, and high-sensitivity C-reactive protein.

### 2.6 Patient follow-up and outcome assessment

Patients (or their relatives) were followed up through specialized clinics or phone calls, with the follow-up period ranging from July 2022 to October 2023. The primary endpoint was the occurrence of major adverse cardiovascular events (MACE) during the follow-up period. MACE included all-cause mortality, cardiac and non-cardiac mortality, and rehospitalization due to myocardial infarction, unstable angina, heart failure, stroke, or subsequent revascularization procedures. All reported events were independently reviewed to evaluate their relationship to the vasculature. Clinical outcomes were defined according to criteria established by the Academic Research Consortium.

### 2.7 Statistical analysis

Continuous variables were presented as mean ± standard deviation (SD) or median (interquartile range), while categorical variables were presented as numbers (percentages). Patients were divided into MACE group and non-MACE group based on the occurrence of MACE. Data comparisons were conducted using Student’s t-test, Mann-Whitney U test, or chi-square test. Pearson or Spearman correlation analysis was employed for assessing correlations. The FAI was dichotomized using receiver operating characteristic (ROC) analysis, with the area under the curve (AUC) and Youden index (defined as % sensitivity + % specificity—1) used to determine the optimal threshold. For cardiovascular event outcomes, univariate and multivariate analyses, including log-rank tests and backward stepwise selection method in Cox proportional hazards regression models, were performed with minimal and fully adjusted models to evaluate predictors of MACE. Model 1 included baseline data; model 2 included model 1 plus FAI; model 3 included model 1 plus HRPC; and model 4 included model 1 plus FAI and HRPC. AUC comparisons among the models were conducted according to the method described by DeLong et al. [20], and the integrated discrimination improvement (IDI) and net reclassification improvement (NRI) were used to compare the reclassification performance of each model. By using the bootstrap method, repeated sampling of the original data is used to generate multiple sample sets, and then the parameters are estimated and confidence intervals are constructed based on these sample sets to evaluate the robustness of the inference method to prevent overfitting. All statistical analyses were performed using MedCalc 20.027 (MedCalc Software, Ostend, Belgium), SPSS 26.0 (IBM Corp., Armonk, NY, USA), and R 4.3.1 (R Foundation for Statistical Computing, Vienna, Austria) software. All probability values were 2-sided, and p values <0.05 were considered statistically significant.

## 3. Results

**Fig 1:**
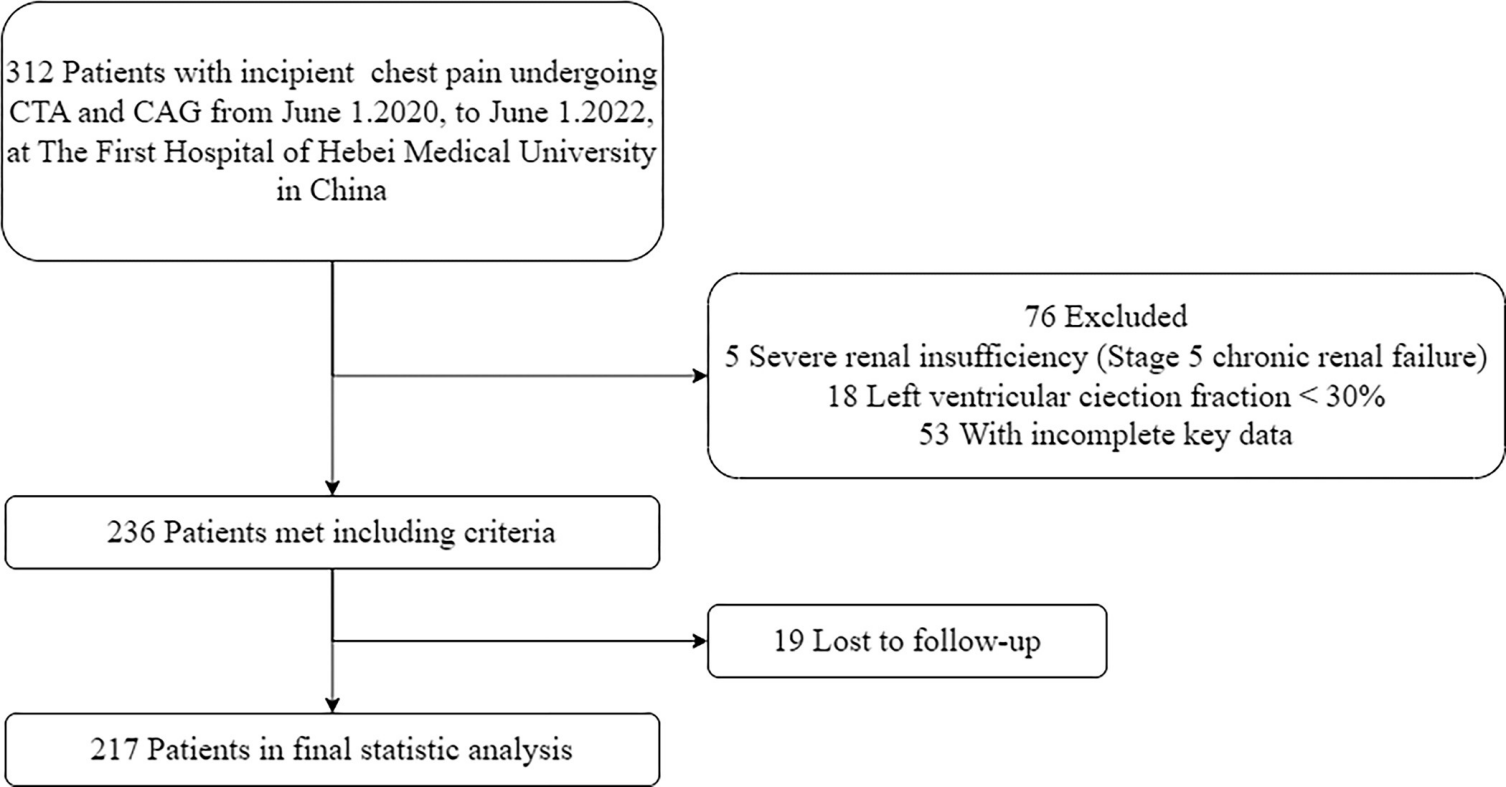
Study population enrollment, exclusion, and inclusion. CTA = computed tomography angiography; CAG = Coronary Angiography.

The patient selection process is shown in Fig 1, and the baseline characteristics of the patients are presented in Table 1. A total of 217 patients were included in this study, with a follow-up period of 15 months. During this period, 48 (22.12%) patients experienced MACE (MACE group), while 169 (77.88%) patients did not experience MACE (non-MACE group). The average age of the patients was 64.95 ± 10.82 years, with 139 (64.1%) being male. The FAI value was -73.48 ± 8.16 HU in the MACE group and -76.73 ± 6.89 HU in the non-MACE group (p = 0.006). The MACE group had a higher proportion of patients with two high-risk plaques compared to the non-MACE group (77.1% vs 58.0%, p = 0.016). Smoking history was more prevalent in the MACE group than the non-MACE group (79.2% vs 62.1%, p = 0.028). The ApoA1 value was 1.31 (1.15–1.53) g/L in the MACE group and 1.20 (1.08–1.33) g/L in the non-MACE group (p = 0.005). The absolute neutrophil count was 3.55 (2.85–4.30) 10^9/L in the MACE group and 4.00 (3.18–4.80) 10^9/L in the non-MACE group (p = 0.042). The MACE group had higher Gensini scores than the non-MACE group (p = 0.014). Regarding high-risk plaques, the MACE group had a higher proportion of positive remodeling compared to the non-MACE group (79.2% vs 62.7%, p = 0.033). The MACE group had a higher proportion of patients with lesions in the RCA than the non-MACE group (43.8% vs 24.3%, p = 0.008). There were no statistically significant differences in the remaining baseline characteristics between the groups (p > 0.05).

**Table 1:** Baseline characteristics of the mace/non-mace groups.

Items	Total	Mace	Non-mace	P-value
n(%)	217	48(22.12)	169(77.88)	
FAI(HU)	-76.01±7.30	-73.48±8.16	-76.73±6.89	0.006
HRPC≥2	135(62.2)	37(77.1)	98(58.0)	0.016
General characteristics				
Age, years	64.95±10.82	65.95±12.44	64.67±10.34	0.470
Male, n (%)	139(64.1)	28(58.3)	111(65.7)	0.349
BMI, kg/m^2^	26.18±3.49	26.56±3.21	26.07±3.57	0.396
Ejection fraction, n (%)	66(62.50–70.50)	66(60.25–69.75)	67(63.00–71.00)	0.475
Cardiovascular risk factors, n (%)				
Diabetes mellitus	54(24.9)	17(35.4)	37(21.9)	0.056
Hypertension	159(73.3)	38(79.2)	121(71.6)	0.296
History of Stroke	58(26.7)	17(35.4)	41(24.3)	0.123
History of Smoking	143(65.9)	38(79.2)	105(62.1)	0.028
History of Drinking	78(35.9)	16(33.3)	62(36.7)	0.669
Family history of CAD	38(17.5)	9(18.8)	29(17.2)	0.798
Laboratory characteristics				
Creatinine, μmol/L	68.70(60.80–80.65)	69.75(61.95–84.13)	68.70(60.30–79.95)	0.615
eGFR, mL/min/1.73m^2	78.09±16.64	76.90±17.86	78.43±16.32	0.574
UA, μmol/L	334.50(282.55–401.40)	343.75(291.00–412.93)	332.30(281.50–398.60)	0.293
LDL-C, mmol/L	2.89±0.81	2.89±0.71	2.89±0.84	0.957
HDL-C, mmol/L	1.02(0.87–1.19)	1.06(0.92–1.21)	1.00(0.86–1.19)	0.288
TG, mmol/L	1.39(0.99–2.00)	1.39(1.13–1.97)	1.37(0.98–2.03)	0.616
TC, mmol/L	4.71(3.88–5.36)	4.74(3.94–5.41)	4.66(3.85–5.38)	0.853
ApoA1, g/L	1.21(1.09–1.38)	1.31(1.15–1.53)	1.20(1.08–1.33)	0.005
ApoB, g/L	0.79(0.64–0.98)	0.82(0.69–1.01)	0.77(0.62–0.96)	0.367
Lp(a), mg/L	142.10(75.55–332.10)	160.00(73.48–285.50)	133.80(75.55–338.50)	0.837
Hs-CRP, mg/L	1.50(0.74–3.08)	1.69(0.74–2.73)	1.49(0.75–3.17)	0.831
Neutrophil count,10^9/L	3.90(3.15–4.71)	3.55(2.85–4.30)	4.00(3.18–4.80)	0.042
Coronary angiogram, n (%)				
Stenosis≥50%	181(83.4)	42(87.5)	139(82.2)	0.388
Stenosis≥70%	124(57.1)	26(54.2)	98(58.0)	0.637
Gensini	18(11–26)	22(14.625–35.5)	17(10–25)	0.014
Target vessel location, n (%)				
LAD	147(67.7)	33(68.8)	114(67.5)	0.866
LCX	56(25.8)	13(27.1)	43(25.4)	0.819
RCA	62(28.6)	21(43.8)	41(24.3)	0.008
Multivessel disease	72(33.2)	20(41.7)	52(30.8)	0.157
HRPC, n (%)				
LAP	62(28.6)	18(37.5)	44(26.0)	0.121
PR	144(66.4)	38(79.2)	106(62.7)	0.033
NRS	75(34.6)	21(43.8)	54(32.0)	0.129
Spotty calcification	128(59.0)	34(70.8)	94(55.6)	0.059
Revascularization	48(22.1)	22(45.8)	62(36.7)	0.251

Dates are presented as mean ± SD, medians with inter quartile ranges or percentage. FAI = fat attenuation index; HU = Hounsfield units; Hs-CRP = high sensitive C-reactive protein; HRPC = high-risk plaque characteristics; BMI = body mass index; eGFR = estimated glomerular filtration rate; CAD = coronary artery disease; LDL-C = low-density lipoprotein cholesterol; HDL-C = high-density lipoprotein cholesterol; TG = triglycerides; TC = total cholesterol; UA = Uric Acid; Lp(a) = lipoprotein (a); ApoA1 = apolipoprotein A1; ApoB = apolipoprotein B; LAD, left anterior descending coronary artery; LCX, left circumflex coronary artery; RCA, right coronary artery; High-risk plaque characteristics: 1) PR = positive remodeling; 2) LAP = low attenuation plaque; 3) NRS = napkin ring sign; and 4) spotty calcification.

### 3.1 Correlation analysis of FAI and HRPC

**Fig 2:**
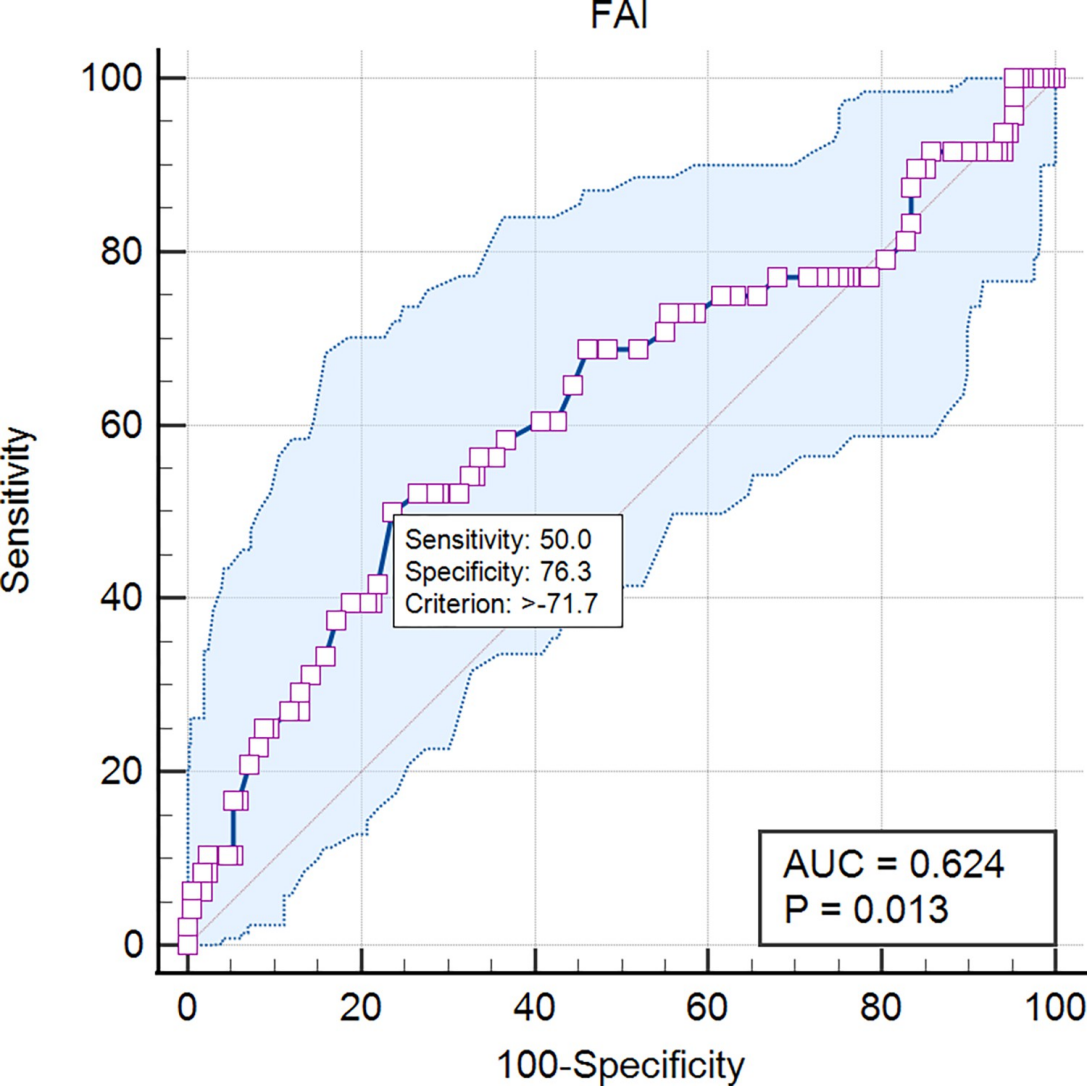
The cutoff value of FAI. FAI = Fat attenuation index.

**Fig 3:**
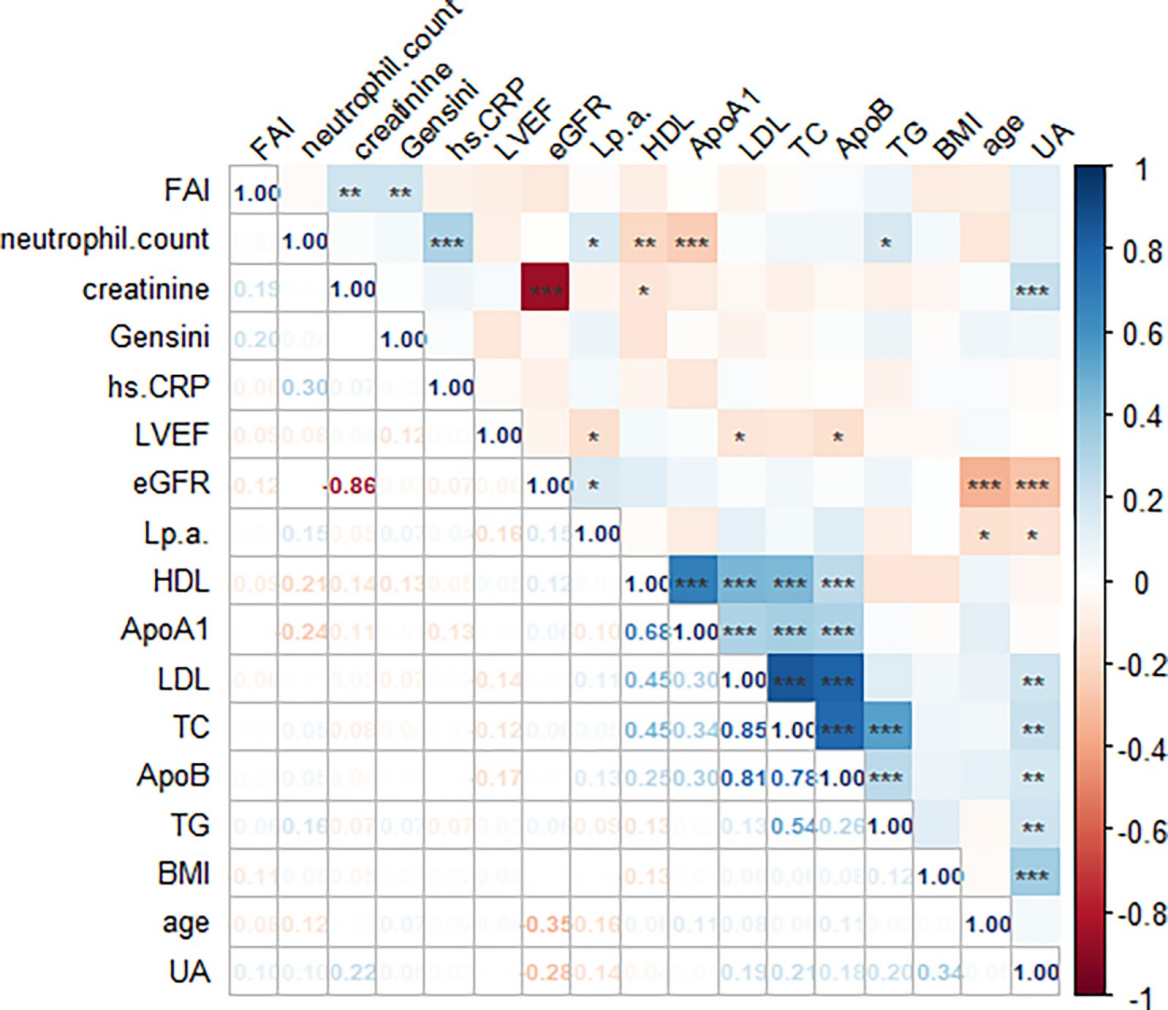
Correlation analysis between continuous variables. FAI = Fat attenuation index; hs-CRP = high sensitive C-reactive protein; BMI = body mass index; LDL = low-density lipoprotein cholesterol; HDL = high-density lipoprotein cholesterol; TG = triglycerides; TC = total cholesterol; UA = Uric Acid; Lp(a) = lipoprotein (a); ApoA1 = apolipoprotein A1; ApoB = apolipoprotein B; eGFR = estimated glomerular filtration rate; LVEF = left ventricular ejection fraction.

**Fig 4:**
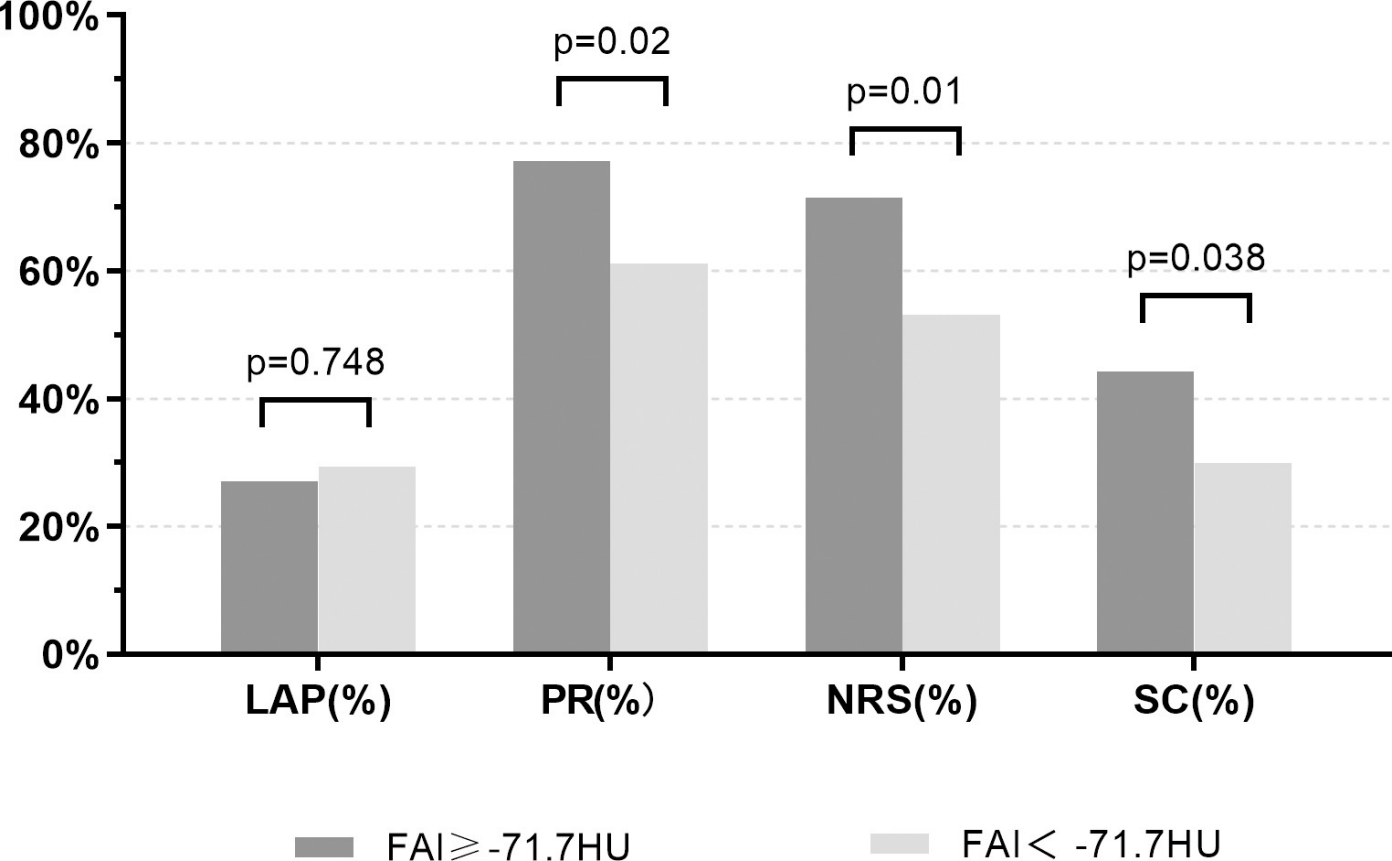
Relationship between FAI and HRPC. FAI = fat attenuation index; HRPC = high-risk plaque characteristics.

The FAI value (mean ± standard deviation) was -76.01 ± 7.30 HU, and the cutoff value calculated by the Youden index shown in Fig 2 was -71.70 HU, with a sensitivity of 50.0% and a specificity of 76.3%. Pearson or Spearman rank correlation coefficients are shown in Fig 3: FAI was positively correlated with creatinine and Gensini score (r = 0.193, p = 0.004; r = 0.198, p = 0.003). The correlation between FAI and hs-CRP as well as absolute neutrophil count was weak (r = -0.062, p = 0.361; r = -0.022, p = 0.751). Regarding high-risk plaque features, as shown in Fig 4, PR, NRS, and SC were more common in the FAI ≥ -71.1 group (77.1% vs 61.2%, 71.4% vs 53.1%, 44.3% vs 29.9%, respectively, all p < 0.05). However, there was no significant difference in LAP between the FAI ≥ -71.1 group and the FAI < -71.1 group (27.1% vs 29.3%, p = 0.748).

### 3.2 Relationship between FAI, HRPC, and coronary artery stenosis

**Fig 5:**
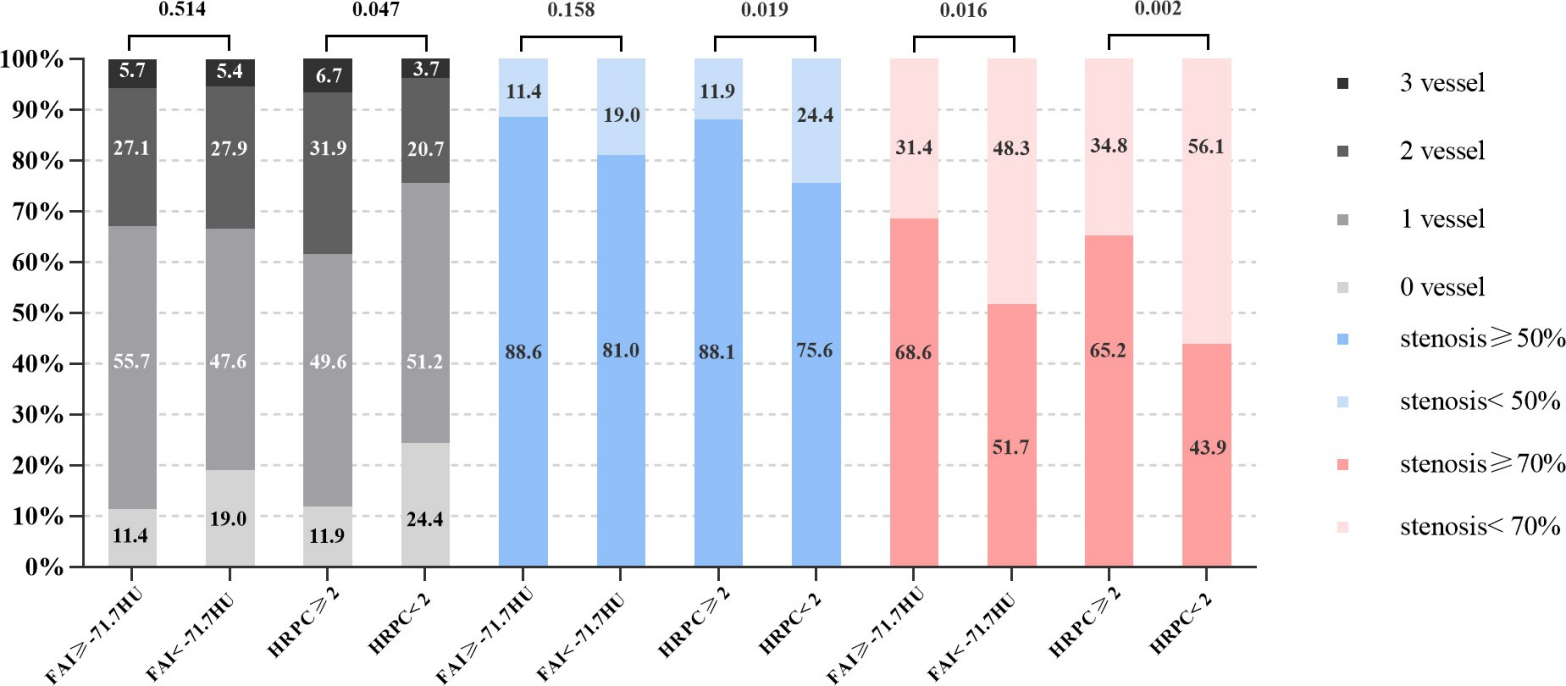
Relationship between FAI, HRPC, and coronary artery stenosis. FAI = fat attenuation index; HRPC = high-risk plaque characteristics.

The relationship between FAI ≥ -71.7 HU, HRPC ≥ 2, and the degree of stenosis is shown in Fig 5. In the FAI ≥ -71.1 group, there were more patients with a stenosis degree ≥ 70% (68.6% vs 51.7%, p = 0.019), and a higher trend of patients with a stenosis degree ≥ 50%, but without statistical significance (88.6% vs 81.0%, p = 0.158), as well as a trend of more multi-vessel disease without statistical significance (p = 0.514). In the HRPC ≥ 2 group, there were more patients with a stenosis degree ≥ 70% and ≥ 50% (65.2% vs 43.9%, p = 0.002; 88.1% vs 75.6%, p = 0.016), and a higher occurrence of multi-vessel disease (p = 0.047).

### 3.3 Prediction of MACE by FAI and HRPC

**Fig 6:**
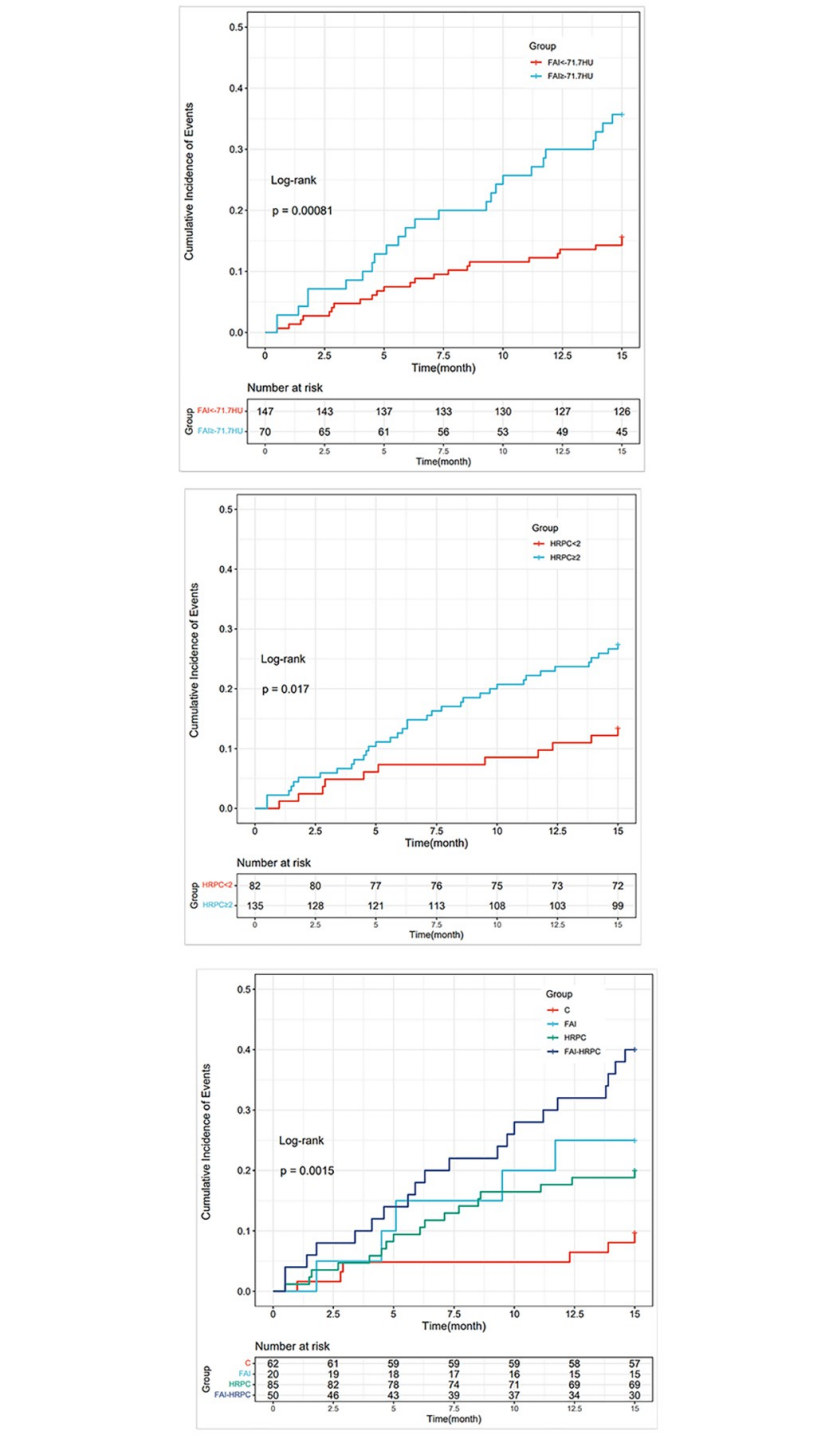
Kaplan-Meier curves for FAI (a), HRPC (b) and the four groups (c). FAI = fat attenuation index; HRPC = high-risk plaque characteristics.

Based on FAI and HRPC, our study further divided the study population into four groups: 1) HRPC ≥ 2 or HRPC < 2 (defined as positive remodeling, low attenuated plaque, punctate calcification, or napkin-ring sign); 2) According to the optimal cutoff value of FAI: FAI ≥ -71.7 HU or FAI < -71.7 HU. The four groups were defined as follows: C = FAI < -71.7 HU and HRPC < 2; FAI = FAI ≥ -71.7 HU and HRPC < 2; HRPC = FAI < -71.7 HU and HRPC ≥ 2; FAI-HRPC = FAI ≥ -71.7 HU and HRPC ≥ 2. Kaplan-Meier curves for FAI, HRPC and the four groups are shown in Fig 6, indicating that FAI and HRPC, either alone or in combination, have an impact on prognosis.

The results of the multivariable Cox analysis, adjusted for age, sex, diabetes, smoking history, ApoA1, uric acid, LDL-C, TG, Gensini score, and eGFR, are shown in Table 2. Compared with the C reference group, the adjusted risk of MACE increased 6.5 times in the FAI-HRPC group (p < 0.001). The HRPC group also had a higher risk compared to the C group (hazard ratio [HR]: 3.30; 95% confidence interval [CI]: 1.24–8.81; p = 0.017).

**Table 2:** Risk of cardiovascular events using the FAI and HRPC groups.

	HR(95%CI)					
	Unadjusted	p-value	Model 1	p-value	Model 2	p-value
C(reference)		0.004		0.001		0.004
FAI	2.852(0.870–9.348)	0.084	2.436(0.718–8.266)	0.153	2.813(0.796–9.946)	0.108
HRPC	2.221(0.875–5.633)	0.093	2.705(1.047–6.986)	0.04	3.302(1.238–8.808)	0.017
FAI-HRPC	4.904(1.968–12.220)	0.001	6.486(2.463–17.081)	<0.001	6.477(2.342–17.911)	<0.001

C = FAI<-71.7HU and HRPC<2; FAI = FAI≥-71.7HU and HRPC<2; HRPC = FAI<-71.7HU and HRPC≥2; FAI-HRPC = FAI≥-71.7HU and HRPC≥2.The log-rank test and backward stepwise selection methods in a Cox proportional hazards regression model was performed; Model 1: adjusted for age, sex, Diabetes mellitus, History of Smoking, ApoA1;Model 2: Model 1 + UA, LDL-C, TG, eGFR, Gensini.

### 3.4 Predictive value of FAI and HRPC combination for prognosis

**Fig 7:**
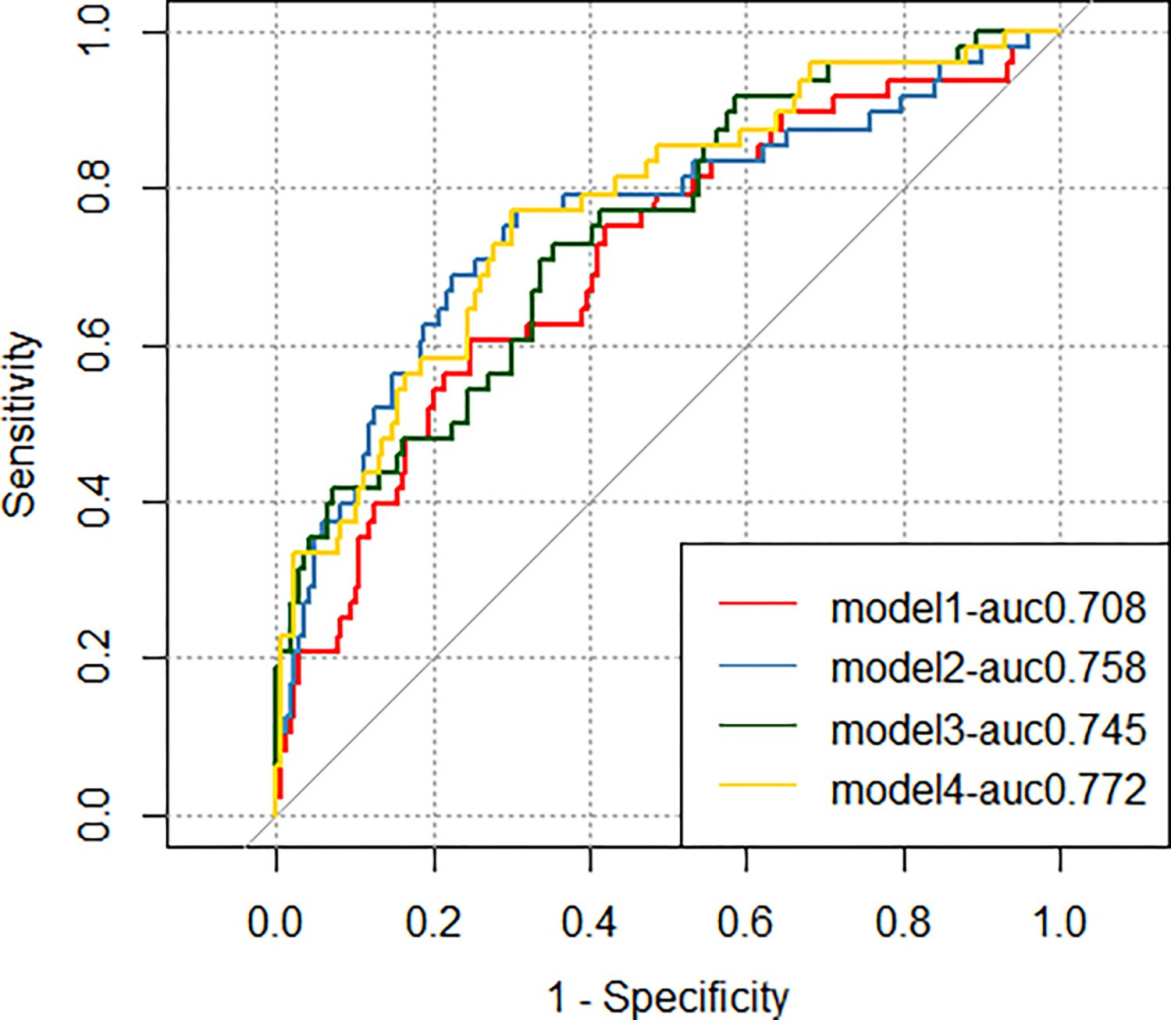
The ROC curves for the four models. Model 1 included baseline data; model 2 included model 1 plus FAI; model 3 included model 1 plus HRPC; and model 4 included model 1 plus FAI and HRPC.

The ROC curves for the four models are shown in Fig 7. The AUC, category-free NRI, and relative IDI values for the four models are shown in Table 3. The inclusion of FAI ≥ -71.7 HU and HRPC ≥ 2 increased the ability to identify adverse cardiovascular events (AUC, 0.772 vs 0.708, p = 0.0465) and the ability to reclassify (NRI, 0.266, p = 0.008; relative IDI, 0.075, p = 0.016). The inclusion of FAI ≥ -71.7 HU or HRPC ≥ 2 alone slightly increased the identification ability (AUC, 0.758 vs 0.708, 0.745 vs 0.708), but the difference was not statistically significant. The combination of FAI and HRPC slightly increased the identification ability compared to FAI or HRPC alone (AUC, 0.772 vs 0.758, 0.772 vs 0.745), but the difference was not statistically significant.

**Table 3:** Additional predictive value provided by FAI and HRPC for predicting MACE.

	C-Statistic	p-value	Bootstrap	Delong	NRI(95%CI)	p-value	IDI(95%CI)	p-value
Original model(reference)	0.708 (0.643–0.767)	p<0.001	0.702 (0.604–0.742)					
Original model+FAI	0.758 (0.695–0.813)	p<0.001	0.729 (0.633–0.777)	p = 0.0653	0.269(-0.058–0.413)	0.108	0.038(-0.005–0.123)	0.096
Original model+HRPC	0.745 (0.681–0.801)	p<0.001	0.721 (0.621–0.753)	p = 0.1871	0.205(-0.016–0.339)	0.076	0.048(0.003–0.107)	0.040
Original model+FAI+HRPC	0.772 (0.710–0.826)	p<0.001	0.745 (0.650–0.782)	p = 0.0465	0.266(0.041–0.419)	0.008	0.075(0.017–0.158)	0.016
Original model+FAI(reference)								
Original model+FAI+HRPC				p = 0.4503	0.189(-0.055–0.325)	0.092	0.037(-0.001–0.089)	0.064
Original model+HRPC(reference)								
Original model+FAI+HRPC				p = 0.2336	0.228(-0.078–0.411)	0.124	0.027(-0.005–0.097)	0.120

NRI = net reclassification index; IDI = integrated discrimination improvement; FAI = fat attenuation index; HRPC = high-risk plaque characteristics. Original model included age, gender, diabetes, history of smoking,UA,ApoA1,Gensini,LDL-C,eGFR,TG.

### 3.5 Subgroup analysis—Revascularized vs. non-revascularized population

**Fig 8:**
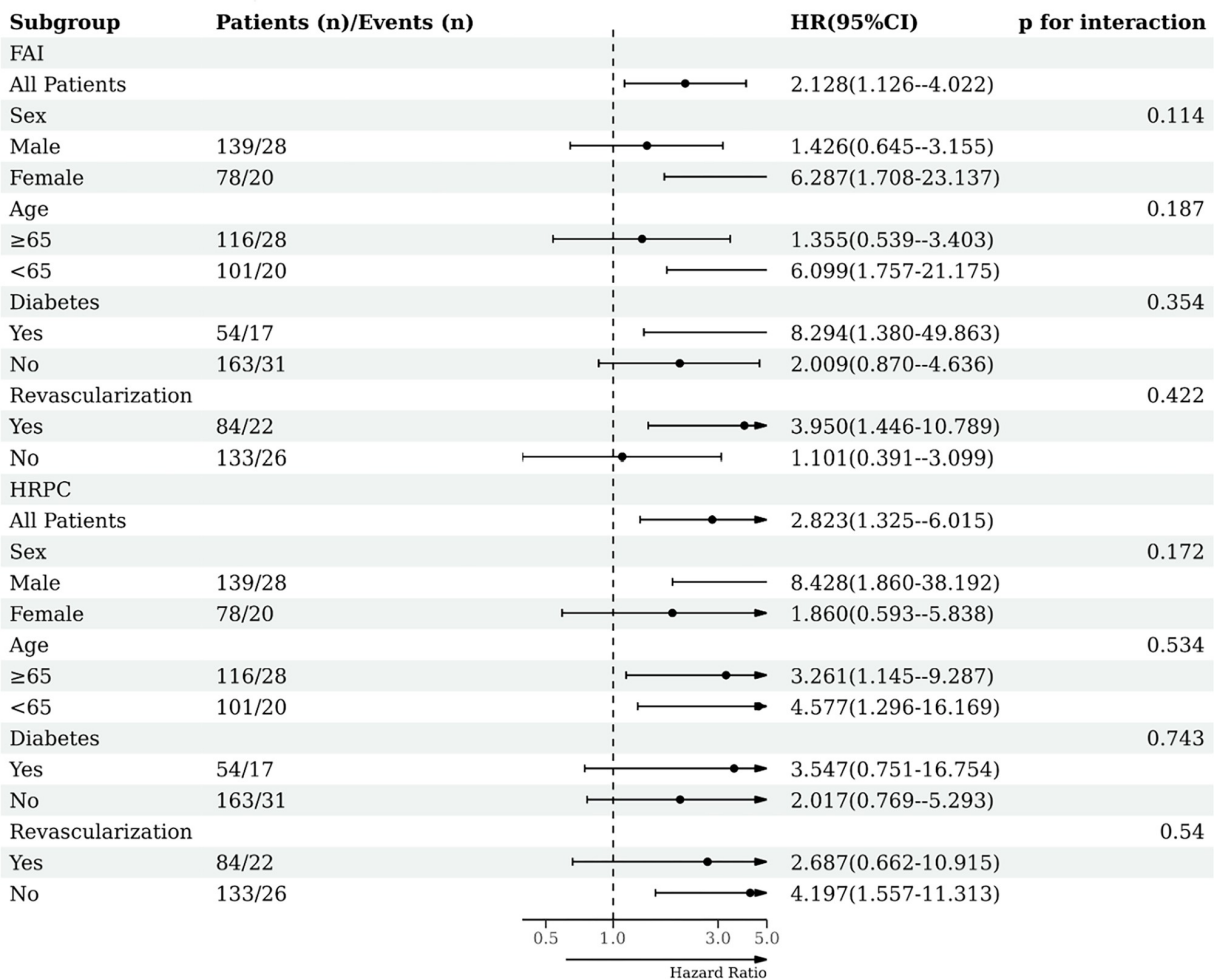
Subgroup analysis—revascularized vs. non-revascularized population.

Further subgroup analysis, as depicted in Fig 8, demonstrates that FAI alone serves as an independent predictor for MACE in the female, <65 years old, diabetes, and revascularized population with hazard ratios of 6.287 (1.708–23.137), 6.099 (1.757–21.175), 8.294 (1.38–49.863), and 3.95 (1.446–10.789) respectively, all p < 0.05. On the other hand, HRPC alone emerges as an independent predictor for MACE in the male, non-revascularized population with hazard ratios of 8.428 (1.860–38.192) and 4.197 (1.557–11.313) respectively, both p < 0.05. Remarkably, irrespective of age, HRPC alone showcases predictive value for the occurrence of MACE.

## 4. Discussion

In our study, we aimed to elucidate the predictive risk of FAI combined with HRPC on the occurrence of MACE in adults with new-onset chest pain. The main findings of this study are as follows: the group with higher FAI values (≥ -71.7 HU) is more prone to high-risk plaques (PR, NRS, and SC). Both FAI and high-risk plaque characteristics are positively correlated with the severity of coronary artery stenosis. Diabetes, smoking history, uric acid, and ApoA1 independently predict the occurrence of MACE. FAI and HRPC alone are associated with prognosis, while the addition of FAI ≥ -71.7 HU and HRPC ≥ 2 increases the ability to identify and reclassify MACE. The diagnostic performance of the combined FAI and HRPC in predicting MACE is slightly higher than that of FAI or HRPC alone, but the difference is not statistically significant.

When the coronary arteries undergo inflammation, the inflammatory signals released by the vascular wall through endocrine and paracrine mechanisms diffuse to the surrounding adipose tissue [21]. These inflammatory signals affect the biological processes of adipocyte differentiation, proliferation, and lipid metabolism, inhibiting local adipogenesis and preventing lipid accumulation. As a result, the composition of adipose tissue around the coronary arteries undergoing inflammation changes, altering the water-to-fat ratio of the adipose tissue [8]. This change in adipose tissue composition can be assessed by coronary artery CT imaging, thus defining the FAI. In the study conducted by Lavi et al., they extensively elucidated how vascular inflammation impacts the local vascular endothelial function, potentially leading to the formation of local narrowings [22, 23]. This finding is consistent with our research results, indicating a significant correlation between high FAI values and more severe vascular inflammation. In fact, high FAI values may serve as a crucial indicator of intensified vascular inflammation, which may further result in more pronounced vascular narrowing. This discovery holds important clinical significance for our understanding of vascular inflammation and its impact on vascular function. In our study, we investigated the cutoff value of FAI and found it to be remarkably close to the values reported previously (-70.10 HU, -71.90 HU) [8, 24]. This outcome suggests that the cutoff value we selected demonstrates high stability and reliability, serving as a vital indicator for evaluating the severity of vascular inflammation. Such consistency provides a reliable foundation for our research and further confirms the potential application of the FAI cutoff value in assessing the degree of vascular inflammation. However, it is worth noting that the cutoff value of FAI remains an ongoing area of investigation. With further research, the optimization and standardization of the cutoff value will enhance the accuracy and reliability of its application. In future studies, through correlational analyses with other relevant indicators and clinical outcomes, we can better validate the efficacy of the cutoff value and explore its potential application in the diagnosis and treatment of vascular inflammation.

The FAI value has been confirmed as an indicator for assessing vascular inflammation through multiple studies. Previous research has indicated a correlation between commonly used biomarkers such as hs-CRP and neutrophil absolute count with vascular inflammation [25, 26]. However, our study findings show no significant correlation between FAI and hs-CRP or neutrophil absolute count, which is consistent with the results of Xu Dai et al [27]. This discovery has prompted us to further contemplate the relationship between FAI and other biomarkers. While hs-CRP and neutrophil absolute count are widely employed to evaluate systemic inflammation [28], we must also consider their limitations in reflecting localized inflammation in coronary arteries. Since coronary artery inflammation typically exhibits a localized distribution, hs-CRP and neutrophils may not accurately capture the inflammatory changes in areas affected by coronary artery disease [29]. This may be one of the reasons why no apparent correlation was observed between FAI and these biomarkers. Additionally, other factors may influence FAI values, such as adipocyte enlargement in patients with insulin resistance in diabetes [30]. It is possible that the relatively small sample size of this study prevented a comprehensive analysis of specific population patterns, necessitating further research with larger sample data. It is worth noting that the consistency of research results contributes to a strengthened understanding of FAI. In line with other study findings, our research deepens our comprehension of FAI. This suggests that FAI may be a unique indicator with distinct biological significance compared to other biomarkers, necessitating further research to uncover its potential diagnostic and prognostic value. In conclusion, although hs-CRP and neutrophils are easily accessible biomarkers, their correlation with FAI is limited. This finding highlights the importance of FAI as an independent indicator and calls for further research to explore its clinical application potential in evaluating vascular inflammation and related diseases. Moreover, this study discovered that for non-AMI patients with initial chest pain, FAI is associated with prognosis, while no correlation was found between hs-CRP or neutrophil absolute count and prognosis. This finding suggests that FAI may serve as a valuable prognostic indicator for specific patient populations, aiding in the assessment of the risk level of disease progression.

Previous studies have already established the strong predictive value of coronary plaque characteristics for patient prognosis [13, 14]. Naya et al. [31] reported no significant correlation between plaque characteristics (including plaque length, composition, or CCTA-derived remodeling index) and ischemia. Furthermore, our research findings demonstrate a positive correlation between plaque characteristics and the degree of coronary artery stenosis, which is consistent with the results of Lee JM et al [32]. This discovery further supports the association between plaque characteristics and coronary artery narrowing, providing important clues for the assessment of coronary artery disease. However, it should be noted that coronary artery stenosis does not necessarily directly result in ischemia in terms of functional significance [33, 34]. This indicates that considering only the degree of stenosis may be insufficient when evaluating clinical outcomes and prognosis of coronary artery disease. Therefore, in order to more accurately adjust for the impact of coronary artery stenosis on prognosis, we introduced the Gensini score, which comprehensively reflects the extent of coronary artery narrowing. By incorporating the Gensini score, we further confirmed that high-risk plaques are independent risk factors beyond the degree of coronary artery stenosis. This suggests that even under similar degrees of stenosis, high-risk plaques may still contribute to more severe clinical outcomes. This finding emphasizes the importance of high-risk plaque characteristics as significant indicators for assessing the risk of coronary artery disease. It is worth noting that the introduction of the Gensini score provides us with a comprehensive approach to evaluating coronary artery disease, enabling a more comprehensive understanding of the impact of plaque characteristics on relevant outcomes. Additionally, future research is still needed to further validate and refine the application of the Gensini score in clinical practice, in order to enhance its accuracy and predictive performance.

Similar to the findings of a CRISP-CT study [35], the group with FAI ≥ -71.7 HU and HRPC ≥ 2 showed a 6.5-fold higher risk of MACE compared to the reference group after adjustment using the Cox model. However, contrary to the findings of the CRISP-CT study, in our research, no statistically significant difference was observed when comparing the FAI ≥ -71.7 HU and HRPC < 2 group with the reference group. This discrepancy may be attributed to the differences in the FAI cutoff value, grouping of high-risk plaques, and the population studied. In the CRISP-CT study, they grouped plaques based on a cutoff of ≥1, whereas in our study, we chose ≥2 for grouping. This difference may be due to a considerable body of existing research confirming that ≥2 plaques may be one of the best predictors of coronary artery events [13, 36]. Additionally, different results may also be related to differences in patient characteristics, duration of follow-up, the number and types of events considered for major adverse cardiovascular events (MACE) definition, and variations in the measurement of coronary heart disease risk factors. Differences in patient characteristics can include age, gender, medical history, and the presence of other relevant diseases. Differences in follow-up duration may lead to variations in the number and types of observed events. Furthermore, variations in the definition of MACE may also exist, including consideration of different types of coronary artery events. Finally, the methods and accuracy of measuring coronary heart disease risk factors may differ across studies. The combined impact of all these factors may contribute to the inconsistencies between our research findings and those of the CRISP-CT study. Despite this, these differences are not meaningless; on the contrary, they remind us to consider the influence of multiple factors comprehensively when interpreting research results and to be mindful of them during study design and interpretation.

Previous studies by Hankun Yan et al. have combined the coronary artery adipose attenuation index (FAI) with high-risk plaque characteristics to analyze ischemic coronary lesions. The results suggest that the addition of FAI and high-risk plaque improves the discrimination of ischemia [24]. According to our research findings, the approach of considering both high-risk plaque characteristics and FAI around the coronary artery performs significantly better in predicting major adverse cardiovascular events (MACE) compared to traditional risk factors, and slightly better than using FAI or HRPC alone. This discovery suggests that the combined approach may be a valuable strategy for assessing in new-onset chest pain adults without acute myocardial infarction (AMI). Although the combined use of FAI and HRPC only achieved a modest improvement in predictive power beyond that achieved by either measure alone, such a small improvement should not be underestimated in the early stages of CAD risk assessment and screening. Specifically, even modest improvements in these domains may be critical in the precise identification of at-risk individuals for the initiation of early preventive interventions. Moreover, given the importance of mitigating the risk of heart disease, such advances, regardless of their magnitude, have the potential to drive more proactive intervention and management strategies that may effectively reduce the likelihood of cardiovascular events in patients. Therefore, it is worth recommending the combined FAI and HRPC assessment in clinical decision-making as an auxiliary tool to enhance the accuracy and comprehensiveness of coronary heart disease risk assessment. By considering multiple factors comprehensively, we can more accurately evaluate a patient’s coronary artery risk and provide more precise predictive outcomes. Furthermore, through subgroup analysis, we found that although different trends were observed in some subgroups, these differences did not reach statistical significance. This indicates the reliability of our findings, suggesting that the combined method of FAI around the coronary artery and high-risk plaque features may have good predictive performance in different subgroups. However, it is important to note that the number of cases in subgroups is relatively small, and further research is needed to determine the optimal parameters or combinations of parameters in different patient populations and lesion subgroups. This will help refine our predictive models and provide more accurate predictive tools for individualized treatment decisions. By conducting larger-scale studies and conducting in-depth analyses on specific populations, we can increase our understanding of the combined approach and optimize its application in different subgroups.

Coronary CTA is one of the most commonly used examinations for patients with new-onset chest pain, as it is convenient, cost-effective, and non-invasive, making it a crucial diagnostic tool [37, 38]. If we fully exploit the role of coronary CTA, we can provide more assistance to clinical diagnosis and treatment without increasing costs. Both FAI alone and high-risk plaques complement traditional risk factors, yet research on their combined correlation remains limited, especially in patients with new-onset chest pain. When FAI or HRPC is used alone, the risk of cardiovascular events may be overestimated or underestimated. Our study determined that the presence of FAI improves the risk prediction of cardiovascular events when HRPC alone or traditional risk factors are used, as demonstrated by ROC analysis, net reclassification index, and integrated discrimination improvement index. Incorporating FAI and plaque characteristics into routine CCTA reports significantly improves risk stratification for patients with new-onset chest pain. This not only saves costs but also better guides clinicians in making subsequent treatment and prevention decisions. It would be interesting to explore whether the use of FFR and other hemodynamic parameters (such as wall shear stress and plaque stress), in addition to coronary CTA-derived plaque characteristics, would yield similar results.

There are several limitations to consider in this study. Firstly, the retrospective design of our study may have subject the results to information and forgetting biases. Data collection in a retrospective design relies on past records and literature, so there may be the possibility of incomplete or inaccurate information. Secondly, potential selection bias in the study may affect the internal validity of the results. For example, sample selection methods may result in a particular type of participant being over-or under-included in the study, thereby affecting the degree to which the results can be generalized. Thirdly, the number of cases is relatively small, leading to a limited number of serious endpoints such as death or myocardial infarction, which prevents the analysis of secondary endpoints. Fourthly, the threshold for FAI and the plaque characteristics were generated from the data in this study. Therefore, the optimal threshold may vary among different populations. Fifthly, this study lacks a comparison with other inflammatory imaging methods, such as positron emission tomography, so the extent of plaque inflammation may not be fully validated in this study. However, this study does confirm the relationship between the analyzed markers and prognosis. Sixthly, our analysis was limited to the four most definite high-risk plaque characteristics (positive remodeling, low attenuation plaque, napkin-ring sign, and spotty calcification) and did not analyze some other minor features. Lastly, the results of this study may not be generalizable to other racial or ethnic groups, because our participants were all Chinese, and our study population focused on individuals with new chest pain rather than acute myocardial infarction, and other types of people, such as stable angina, were not analyzed. Future research can be compared and explored among different types of people. To comprehensively understand the performance and predictors of CHD risk assessment in different populations. Such research will help promote the development of personalized medicine and provide better prevention and treatment strategies for different types of patients.

## 5. Conclusions

In conclusion, both the peri-coronary fat index and high-risk plaques are significantly associated with the risk of clinical events. Combining peri-coronary fat index and plaque vulnerability, considering the differential prognostic significance of FAI and HRPC, may provide better risk stratification than using either group alone, potentially supporting improved cardiovascular risk management with minimal cost increase.

## Abbreviations

CHD: Coronary artery disease
FAI: Fat attenuation index
HRPC: High-risk plaque characteristics
CCTA: Coronary computed tomography angiography
CAG: Coronary angiography
AMI: Acute myocardial infarction
MACE: Major adverse cardiovascular events
